# Concomitant Neck and Lung Masses Post Dental Procedure—A Potential Novel Presentation of the *Cellulosimicrobium* Species in Humans

**DOI:** 10.3390/idr17050103

**Published:** 2025-08-22

**Authors:** Kevin M. Sheehan, Geraldine Moloney, Olive Murphy, Paul Ryan, Triona Hayes, Madeleine R. Harney, Michael Harney, Oisin O’Connell

**Affiliations:** 1 Department of Radiology, University of Galway, H91 V4AY Galway, Ireland; 2 Department of Infectious Diseases, Galway University Hospital, H91 YR71 Galway, Ireland; 3 Department of Microbiology, Bon Secours Hospital Cork, T12 DV56 Cork, Ireland; 4 Department of Pathology, Bon Secours Hospital Cork, T12 DV56 Cork, Ireland; 5 Department of Health Sciences, University of Missouri, Columbia, MO 65211, USA; 6 Department of Ear Nose and Throat Surgery, Bon Secours Hospital Cork, T12 DV56 Cork, Ireland; 7 Department of Pulmonology, Bon Secours Hospital Cork, T12 DV56 Cork, Ireland

**Keywords:** *Cellulosimicrobium*, *Oerskovia*, *cellulans*, *funkei*, *terrum*, *xanthineolytica*, *turbata*

## Abstract

**Background:*** Cellulosimicrobium*, formerly known as the *Oerskovia* genus, is a Gram-positive organism known for its characteristic bright yellow colonies. While abundant in nature, it is very rarely linked to pathogenesis in humans. While there is no classical presentation for *Cellulosimicrobium*-associated infections, cases tend to be foreign body-related or involve immunocompromised patients. Rates of *Cellulosimicrobium*-associated infections have been hypothesised to rise in the future, due to rising numbers of immunocompromised patients in the community and increasing usage of foreign bodies such as prostheses and long-term catheters. Existing technical difficulties regarding misidentifying cultures as other species (often other coryneforms) may also play a significant role in the low number of documented cases, and this may change in the near future with diagnostic advancements such as whole genomic sequencing. **Case Presentation:** A 57-year-old immunocompetent Irish male presented with concomitant neck and lung masses. Notably, this was found to be directly following a recent dental procedure. During extensive investigations, *Cellulosimicrobium* was isolated from biopsied lung tissue using 16S ribosomal ribonucleic acid gene-sequencing analysis. The patient was treated with long-term oral amoxicillin and safely discharged, with both masses showing measurable reductions in size on subsequent imaging. **Conclusions:** Should *Cellulosimicrobium* represent the causative pathological organism in this case, then we believe this to represent a potential novel documented presentation of the organism’s pathogenesis in humans. We provide detailed discussion surrounding the successful management of this patient and the evaluation of the evolving differential diagnosis throughout this case.

## 1. Introduction

*Cellulosimicrobium*, formerly known as the *Oerskovia* genus prior to its widely accepted reclassification by Schumann et al. [1], consists of Gram-positive, branched organisms that are commonly found in soil and are known for the characteristic bright yellow colour of their colonies [2]. Three varieties of these coryneforms exist: *C. cellulans*, *C. funkei* and *C. terrum*. Of these three, *C. cellulans* (previously *O. xanthineolytica*) and *C. funkei* (previously *O. turbata*) have, in rare cases, been attributed to pathogenesis in humans [3,4].

Little work has been published documenting *Cellulosimicrobium*-associated infections. A literature review by Rivero et al. in 2019 identified only 43 cases of infections in humans caused by this organism (along with an additional case that they themselves describe) [3]. A more recent literature review by Ioannou et al. identified only 40 cases of infections attributable to *Cellulosimicrobium* [5]. It has been postulated that difficulties regarding misidentifying cultures of the species as other coryneforms (with subsequent dismissal as skin flora) may also have a part to play in the scarcity of reported cases [6,7].

There is no “typical” presentation in the existing literature, and presentations include manifestations such as central venous catheter-related bacteraemia, peritonitis, endocarditis, meningitis, endopthalmitis, pneumonia, soft tissue infections, osteomyelitis, suppurative arthritis, pyonephrosis, axillary abscesses and bacteraemia of unknown source [3,5]. However, cases tend to be foreign body-related or involve patients who are immunocompromised [3,4,6]. Rivero et al. deemed 26 (60%) of their described cases to have underlying immune dysfunction [3]. Furthermore, 29 (67%) of cases were foreign body-related [3]. Ioannou et al. also identified a large proportion of potentially foreign body-associated cases [5]. Six (15%) of patients underwent surgery within three months of *Cellulosimicrobium* isolation. Eleven (27.5%) patients had a central venous catheter in situ, six (15%) were receiving dialysis, and four (10%) had cardiac valve prostheses [5]. This is in concordance with the findings of Rivero et al. Interestingly, Ioannou et al. identified vancomycin to have the lowest rate of antimicrobial resistance (0 of 29 cases) [5].

Given the paucity of described occurrences, we present one such case in which *Cellulosimicrobium* was isolated to add to the small body of existing literature on *Cellulosimicrobium*-associated infection in humans, with the aim of helping to improve our understanding of its presentation in human disease.

## 2. Case Presentation

A 57-year-old Irish man with a pre-existing background history of coronary artery disease, hypertension, dyslipidaemia and a 60 pack-year smoking history presented to hospital with a left-sided painless neck mass, a dry cough, and decreased appetite. Notably, this was directly preceded by a dental procedure within the previous month. The patient also reported that the timing of symptom onset was accompanied by left-sided dental pain, the side on which the recent procedure had taken place. On examination a firm, fixed, left-sided non-tender neck mass was appreciable; however, the remainder of the physical examination on admission was unremarkable. The patient denied any other constitutional symptoms such as weight loss, lethargy, night sweats, etc.

Haematological investigations on admission revealed a normocytic, normochromic anaemia, with a haemoglobin of 12.5 g/dL (range 14–18). The white blood cell count was marginally elevated at 10.2 × 10.9/L (4–10); however, neutrophil, lymphocyte, and eosinophil values were within normal ranges. Urea and electrolytes were unremarkable. The erythrocyte sedimentation rate (ESR) was notably raised, however, at over 120 mm/HR (<14). C-reactive protein (CRP) was assessed two days later and found to be 58.5 mg/L (<10). No microorganisms were grown via peripheral blood cultures.

**Figure 1:**
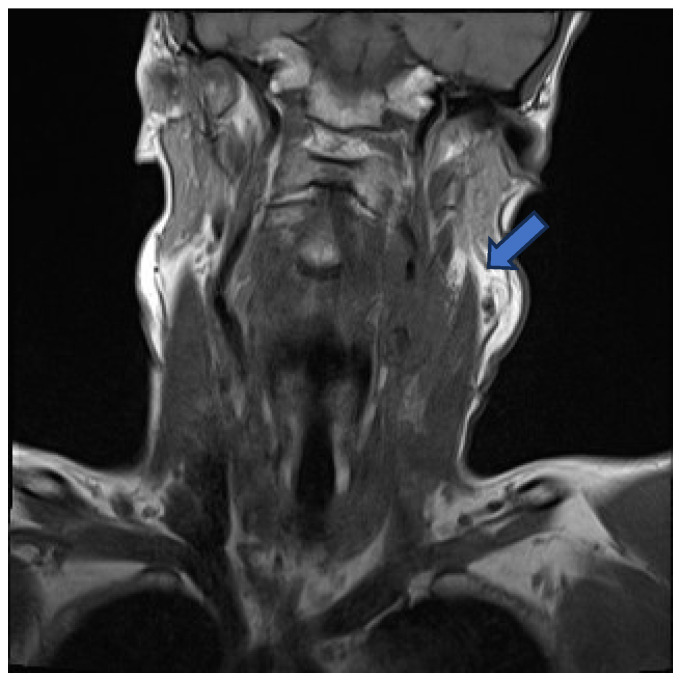
MRI—mass encasing the left common carotid, carotid bulb, and internal carotid arteries (blue arrow).

Initial contrast-enhanced computed tomography (CT) neck revealed a large left-sided neck mass, measuring 3.5 by 4.6 cm. Further characterisation by contrast-enhanced magnetic resonance imaging (MRI) showed the neck mass to be encasing the upper common carotid artery, the carotid bulb, and the internal carotid artery on the left side. This mass was also compressing the left internal jugular vein, occluding it and resulting in thrombosis (Figure 1).

**Figure 2:**
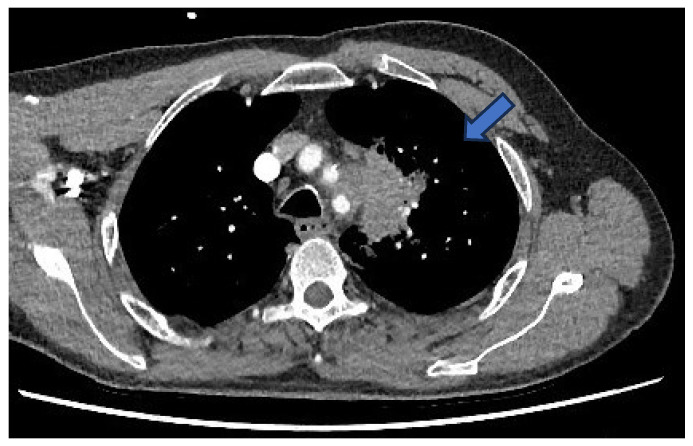
CT—left-sided lung mass (blue arrow) invading into the mediastinum, involving the aortic arch and proximal descending aorta.

During further investigation, contrast-enhanced CT of the thorax, abdomen, and pelvis revealed a 5.5 cm mass in the upper lobe of the left lung (Figure 2). Notably, this mass appeared to invade through tissue planes, encroaching into the mediastinum and involving the arch and proximal descending aorta. There were no other lung findings or suspicious bony lesions.

Ultrasound-guided core biopsies of the neck mass showed a reactive fibroinflammatory proliferation, comprising elongated spindle cells, along with mixed acute and chronic inflammatory cells, and no evidence of neoplastic disease. Spindle cells were negative for cytokeratin, trefoil factor 1 (TFF-1), cytokeratin 5/6 (CK56) and tumour protein p63 on immunohistochemistry. Core biopsies of adjacent cervical lymph nodes were also benign. 

CT-guided percutaneous biopsy of the lung mass was then performed, and again no evidence of malignancy was identified. Biopsies contained both acute and chronic inflammatory cells, in association with a with fibroblast-like spindle cell proliferation. This fibro-inflammatory picture was noted to be similar in appearance to the neck mass core biopsy. Notably, however, Gram-positive rods were isolated from the lung biopsy tissue. Initially described as organisms resembling *Actinomyces*, they were later identified as the *Cellulosimicrobium* species using 16S ribosomal ribonucleic acid (rRNA) gene-sequencing analysis of the biopsied tissue.

Differential Diagnosis: Given the complex nature of this case, the differential diagnosis was broad and is constantly evolving over time. On initial presentation, prior to biopsy, the initial impression was of a likely metastatic lung neoplasm. Indeed, the possibility of two separate neoplastic processes in both the neck and lung was also actively considered. The inability to identify evidence of malignancy on multiple biopsies necessitated the consideration of alternative diagnoses.

Due to the lack of evidence suggestive of neoplastic disease, a wide array of infective causes were considered during the investigative process. Initially, due to the presenting neck mass, combined with the subsequent jugular vein occlusion on imaging, Lemierres disease was also briefly considered; however, this theory was discarded due to the presence of a concomitant lung mass as well as the inability to isolate the classically causative anaerobic organism, *Fusobacterium necrophorum*. *Nocardia* and *Actinomyces* were also considered given the masses’ invasion through tissue planes seen on radiological imaging; however, both prolonged cultures and 16S sRNA gene-sequencing analysis were negative. Given their populational prevalence, *Mycobacterium tuberculosis* infection and other granulomatous diseases such as Sarcoidosis were also considered; however, no granulomata were identified despite multiple biopsies. QuantiFERON and acid-fast bacillus testing was also negative. Given the atypical nature of the presentation, fungal pathologies such as *Coccidioiodomycosis* and *Blastomycosis* were also considered, then disregarded after they could not be isolated in numerous cultures, as well as biopsies.

An important differential to acknowledge is that of inflammatory pseudotumours (IPTs). IPTs represent a rare heterogenous disease process known by several terms, including inflammatory myofibroblastic tumour, pseudosarcoma, myxoid hamartoma, fibrous xanthoma, plasma cell granuloma, and inflammatory myofibrohistiocytic proliferation [8]. The cause is unknown, and multiple hypotheses have been proposed in an attempt to explain its aetiology. These include attributing it to a true primary neoplasm; an immune-mediated response to various infectious organisms (e.g., Epstein–Barr virus (EBV); *Mycobacterium*; *Nocardia*; *Actinomyces*; *Escherichia coli*; *Klebsiella*; human immunodeficiency virus (HIV)) [8,9]; inflammatory response to a causative low-grade neoplasm [10]; trauma; and immunoglobulin G4 (IgG4)-associated disease [9]. Histopathological findings regarding these space-occupying lesions are varied, including spindle cell proliferation, acute and chronic inflammatory changes (e.g., lymphocytes and plasma cells), myofibroblasts, fibroblasts, collagen and granulation tissue [8,9,10,11]. IPTs can vary widely in their location, but are largely found in the viscera or soft tissues [10].

Another inflammatory condition considered in the differential was that of IgG4-related disease (IgG4-RD). This fibroinflammatory process can manifest in multiple systems and the majority of cases see multiple focal areas involved at the time of presentation [12]. Specific to this case, IgG4-RD can manifest as lung lesions, some of which present as solid lung nodules [13,14]. However, it should be noted that the pancreas and salivary glands are the most commonly affected sites [12]. While a fibroblastic and spindle cell proliferation was identified, many associated features such as obliterative phlebitis, storiform pattern fibroblastic proliferation, an abnormally raised ratio of IgG4-positive cells to IgG-positive cells, and lymphoplasmocytic inflammation with eosinophil abundance were not identified on histopathological examination of core biopsies. Furthermore, serum concentrations of IgG4 were not noted to be abnormally elevated.

Management and Follow-up: Given the complex nature of this presentation, management of this case was ever-evolving with the differential and was multi-disciplinary in nature. Biopsying required the consultation of numerous services such as otolaryngology and radiology. Multiple sub-specialised pathologists consulted on the interpretation of biopsies.

Microbiological input was a key factor for ongoing guidance in this case. As the differential evolved, so too did the antibiotic regimen. Initial antibiotic therapy consisted of intravenous ceftriaxone and metronidazole when Lemierre’s was suspected. This regimen was later changed to empiric tuberculosis treatment, as well as meropenem to cover *Nocardia* (given the invasion through tissue planes seen on radiological imaging). Following the identification of Gram-positive rods described as resembling *Actinomyces* from the lung biopsy tissue, high-dose intravenous penicillins were used. This, in turn, coincided with the presenting neck mass decreasing in size on clinical examination. It is important to note that steroids were not administered in conjunction with this antibiotic therapy. Given the growth of Gram-positive rods resemblant of *Actinomyces* as well as the invasion through tissue planes on imaging, the patient was discharged with a prescription of 1 g of oral amoxicillin to be taken three times daily for a year to treat *Actinomyces*. This was tolerated without any undue side effects. This year-long course was continued following later identification of the *Cellulosimicrobium* species from lung biopsy tissue.

Due to compression of the left internal jugular vein by the neck mass and consequent thrombosis, haematology was closely involved in care. Initially commenced on low-molecular-weight heparin on admission, this was changed to 5 mg apixaban twice daily for four months following discharge. This was subsequently reduced to 2.5 mg twice daily and continued for six months. Follow-up CT and Doppler ultrasound scans noted continued thrombosis of the external jugular vein, but that good collaterals had developed. The impression was that this vessel was chronically thrombosed and stable in nature, and so anticoagulation was discontinued.

Following discharge, the patient has remained well despite some residual neck discomfort. On clinical examination, it has been noted that the neck swelling has decreased in size. This was confirmed on follow-up imaging and CT thorax also showed a reduction in the size of the lung mass. Follow-up inflammatory markers were also shown to be reduced. The patient remains in regular outpatient follow-up.

## 3. Discussion

As previously stated, few cases of *Cellulosimicrobium*-associated infections have been documented in the existing literature. Rising numbers of immunocompromised patients in the community, as well as increasing usage of prostheses, long-term catheters, etc., may pose a risk of increasing numbers of *Cellulosimicrobium* infections in humans going forward. Technical difficulties such as misidentification of cultures as other coryneforms likely also have a part to play in the scarcity of documented cases [6,7]. Therefore, ongoing utilisation of technological advancements in diagnostics such as whole-genomic sequencing and matrix-assisted laser desorption/ionisation time-of-flight mass spectrometry may also lead to future increases in *Cellulosimicrobium* isolation. This case, for example, utilised 16S RNA sequencing, a diagnostic technique in keeping with cases described in the literature [6].

Given the discussed potential for future increases in *Cellulosimicrobium*-associated infections, it becomes increasingly important that cases are documented. Thus, our case adds to the small body of existing literature. Following an inability to identify a similar presentation, we believe this case represents a potential novel presentation of *Cellulosimicrobium* pathogenesis in humans. Given the abundance of iatrogenic seeding of the organism in the existing literature, we believe this case was likely attributable to the patient’s recent dental procedure.

However, it would be remiss not to acknowledge the diagnostic uncertainty surrounding this case (discussed at length). An important highlighted differential is that of IPT. However, there are a number of reasons we have attributed the presentation to *Cellulosimicrobium*. First and foremost, the isolation of the organism on lung tissue biopsy using 16S rRNA gene-sequencing analysis has been well documented as an effective diagnostic tool [6]. Other reasons include the timing of presentation in relation to a recently preceding dental procedure, as well as the reduction of lesion sizes, and normalisation of ESR and CRP with antibiotic therapy.

Indeed, even if this presentation is attributable to IPT, as mentioned previously, one hypothesis regarding the aetiology of IPTs is as a response to various infectious organisms. Suspected organisms include *Actinomyces*, to which *Cellulosimicrobium* is closely related, belonging to the *Actinomycetales* order. This may, therefore, represent a case of *Cellulosimicrobium*-related IPT (of which we have been unable to find a documented case of in the existing literature).

## 4. Conclusions

*Cellulosimicrobium* is rarely isolated as the causative organism in infections in humans. These cases are most commonly seen in immunocompromised hosts or are foreign body-related. We present one such potential case to add to the small body of existing literature with the aim to help better our understanding of *Cellulosimicrobium*-related pathogenesis in humans. We believe that this case represents a novel form of presentation of the *Cellulosimicrobium* species in humans.

## Data Availability

Pertinent data has been included in the article. Further inquiries can be directed to the corresponding author.
